# A GntR family transcription factor positively regulates mycobacterial isoniazid resistance by controlling the expression of a putative permease

**DOI:** 10.1186/s12866-015-0556-8

**Published:** 2015-10-16

**Authors:** Jialing Hu, Lei Zhao, Min Yang

**Affiliations:** National Key Laboratory of Agricultural Microbiology, Center for Proteomics Research, College of Life Science and Technology, Huazhong Agricultural University, Wuhan, 430070 China; College of Life Science and Technology, Huazhong Agricultural University, Wuhan, 430070 China

**Keywords:** Mycobacteria, GntR, Isoniazid, Permease

## Abstract

**Background:**

Bacteria use transcriptional regulation to respond to environmental stresses. Specifically, exposure to antibacterial drugs is deemed to be an atypical stress, and altering transcriptional regulation in response to such stress can increase bacterial drug resistance. However, only a few transcription factors that regulate drug resistance have been reported.

**Results:**

In the present study, a GntR family transcription factor, encoded by the *MSMEG_0535 (Ms0535)* gene, was shown to be an isoniazid (INH) resistance regulator in *Mycobacterium smegmatis.* When the *Ms0535* gene was overexpressed, cells showed a significant increase in INH resistance. First, the interaction between Ms0535 and its own promoter was determined, and a conserved 26-bp palindromic DNA binding motif was identified using electrophoretic mobility shift and DNaseI footprinting assays. Second, quantitative reverse transcription-PCR assays showed that Ms0535 acted as a transcriptional activator, and positively regulated its own expression, as well as that of a permease encoded by the *MSMEG_0534 (Ms0534)* gene. Similar to the case for the *Ms0535* gene, a recombinant *Ms0534*-overexpressing strain also exhibited increased INH resistance compared with the wild-type strain. Furthermore, we showed that *Ms0535* and *Ms0534* deletion strains were more sensitive to INH than the wild-type strain. Interestingly, overexpressing *Ms0534* in the *Ms0535* deletion strain enhanced its INH resistance. In contrast, the *Ms0534* deletion strain was still sensitive to INH even when *Ms0535* was overexpressed. These findings suggest that Ms0534 is an effector protein that affects INH resistance in *M. smegmatis*.

**Conclusions:**

In summary, the GntR transcriptional regulator Ms0535 positively regulates INH resistance by transcriptionally regulating the expression of the Ms0534 permease in M. smegmatis. These results improve our understanding of the role of transcriptional regulation in INH drug resistance in mycobacteria.

**Electronic supplementary material:**

The online version of this article (doi:10.1186/s12866-015-0556-8) contains supplementary material, which is available to authorized users.

## Background

Transcriptional regulation plays an important role in the bacterial response to environmental stresses. Antibacterial drugs are deemed to be atypical stressors, and changes in transcriptional regulation can increase bacterial drug resistance [1]. When exposed to such drugs, bacteria can undergo complex responses, such as inactivating the targets, modifying the drugs, or expressing drug efflux system proteins. Today, more than 20 transporter proteins have been found to be associated with tetracycline efflux systems [2]. Bacterial drug efflux pumps can efflux drugs out of the cell, thereby lowering the drug concentration, and up-regulating the expression of membrane drug efflux pump proteins is the key mechanism by which bacteria increase their drug resistance [3]. Five different categories of drug transporters have been investigated: the ATP-binding cassette (ABC) superfamily, the major facilitator superfamily (MFS), the multidrug and toxic compound extrusion (MATE) family, the small multidrug resistance (SMR) family, and the resistance/nodulation/cell division (RND) family [2].

MFS members are widespread, and they play important roles in drug transport in organisms ranging from bacteria to humans [4]. The MFS was first found to transport glucide, and, subsequently, drugs, phosphates, and oligosaccharides [5]. An MFS permease is directly involved in the bacterial drug efflux process [4]. Some bacterial transcription regulatory proteins, such as *Bacillus subtilis* BmrR, *Staphylococcus aureus* QacR, and *Mycobacterium smegmatis* LfrA, can regulate the expression of drug transporters by acting either as transcriptional repressors or activators of drug efflux genes [6–8]. However, our knowledge of the transcriptional factors that regulate anti-mycobacterial drug transporter-related genes is still limited.

*Mycobacterium smegmatis* is a relatively fast-growing and non-pathogenic mycobacterium, and it is frequently used as a model organism to study the gene regulatory mechanisms of *M. tuberculosis* [9, 10]*.* Notably, the genome of *M. tuberculosis* encodes at least 20 possible drug efflux transporters [11]. Some of these transporter proteins play a role in mycobacterial resistance to isoniazid (INH), rifampicin (RIF), tetracycline, and other antibiotics [12–14]. In comparison with *M. tuberculosis, M. smegmatis* has more drug efflux systems and transcription factors; however, the mechanisms by which they are regulated are largely unknown.

GntR family transcription factors, which are named after a gluconate operon repressor in *B. subtilis*, are widely distributed in bacteria [15, 16]. GntR members contain a conserved N-terminal helix-turn-helix (HTH) domain for DNA-binding and diverse C-terminal domains [15]. The variable C-terminal domain provides the basis for their classification into six subfamilies. Except for Ms2173, a global Cu^2+^-responsive transcription factor [17], members of the GntR/FadR subfamily have been rarely reported in *M. smegmatis*. GntR family members usually function as transcriptional repressors, although some of them are transcriptional activators. For instance, *Serratia marcescens* PigT [18], *Enterococcus faecalis* CitO [19], FadR [20], and McbR/YncC [21] from *Escherichia coli*, NorG from *Staphylococcus aureus* [22, 23], HpxS from *Klebsiella pneumoniae* [24], and *B. subtilis* GabR [25] were shown to act as transcriptional activators or repressor/activators.

In this study, we have shown that a GntR family transcription factor, encoded by the *Ms0535* gene, plays a role in *M. smegmatis* drug resistance. Ms0535 specifically bound its own promoter by recognizing a 26-bp palindromic sequence motif that is separated by four nucleotides. It regulates *M. smegmatis* INH resistance by functioning as a transcriptional activator that regulates the expression of the MFS permease gene *Ms0534*, which is located in the same operon. This is the first study to clarify the regulatory mechanism of the transcriptional factor Ms0535 and its role in *M. smegmatis* INH resistance.

## Results

### *Mycobacterium smegmatis* Ms0535 potentially contributes to mycobacterial INH resistance

To identify potential transcription factors that regulate drug resistance in *M. smegmatis*, we screened a transcriptional regulator library by spotting these recombinant strains onto plates containing INH (20 μg/mL). About 500 predicted regulatory genes in the genome of the *M. smegmatis* strain mc^2^155 (Genbank accession number CP000480) were cloned into pMV261, and the recombinant plasmids were transformed into *M. smegmatis*. Transformant strains were spotted onto 7H10 medium containing 20 μg/mL INH. The recombinant plasmids were isolated from the INH-resistant *M. smegmatis* transformants, which enabled the positive regulator genes to be characterized by sequencing.

**Fig. 1:**
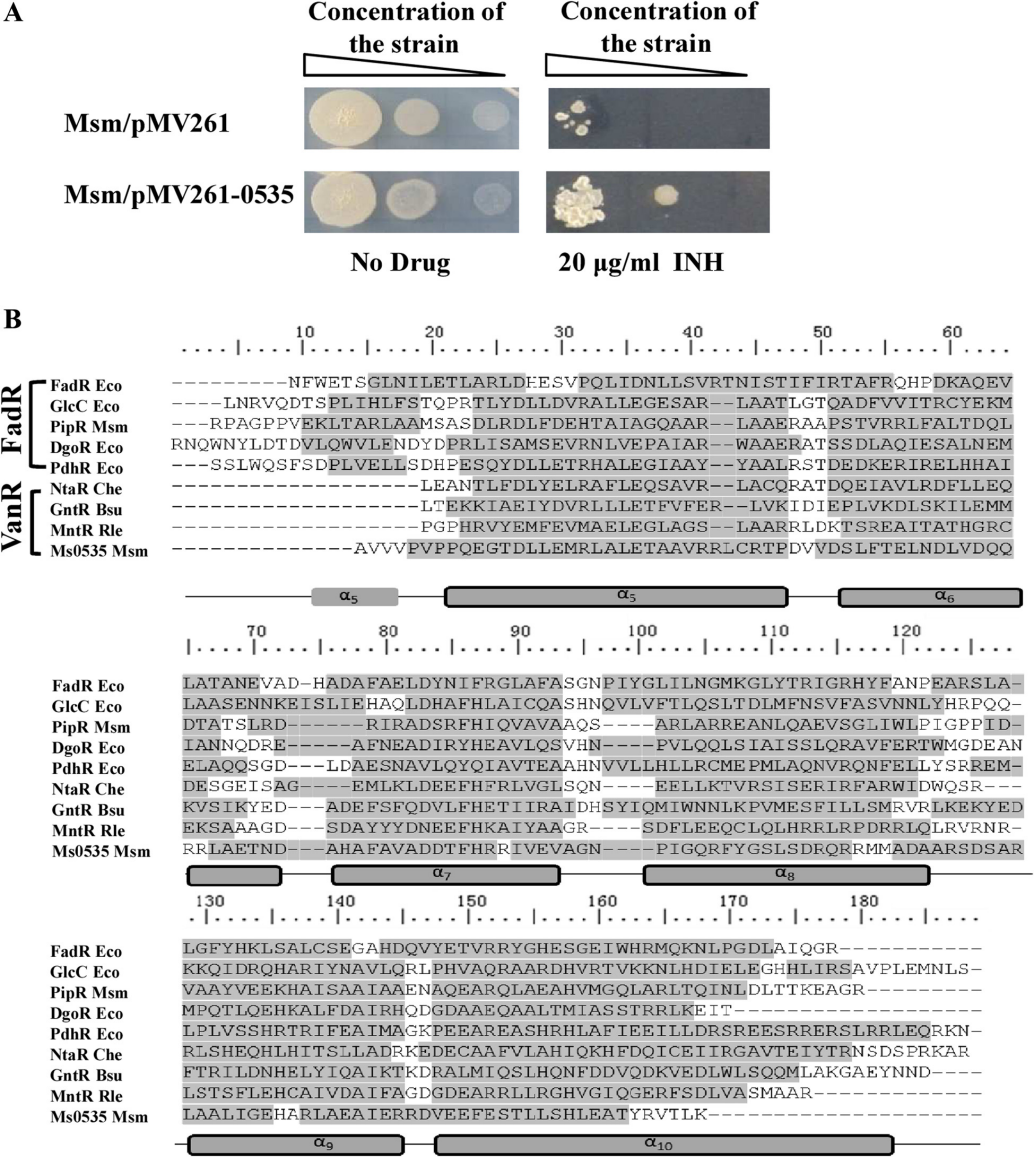
The effect of Ms0535 on the drug resistance of *M. smegmatis* and a sequence alignment of the Ms0535 C-terminal domain. (**a**) Isoniazid (INH)-resistance of the *M. smegmatis* (Msm) strain overexpressing Ms0535. Msm/pMV261 and Msm/pMV261-*Ms0535* were grown on medium containing 30 μg/mL kanamycin for 36 h. Then, different concentrations of freshly grown bacteria were streaked onto 7H10 plates containing kanamycin (30 μg/mL) and INH (20 μg/mL). The control plate did not contain INH. (**b**) Analysis of the structural characteristics of Ms0535. The Ms0535 amino acid sequence was analyzed using the online tool SWISS-MODEL. The N-terminal region of Ms0535 contains a winged helix-turn-helix GntR DNA-binding domain, while its C-terminus contains a domain that is similar to that of the FCD family. Amino acid residues present in α-helices are highlighted in gray, and spaces in the consensus sequence denote insertions within the alignment

A hypothetical transcriptional factor, encoded by the *Ms0535* gene, was isolated as a potential contributor to INH resistance in *M. smegmatis*. As shown in Fig. 1a, the mycobacterial strain transformed with pMV261-*Ms0535*, which overexpresses *Ms0535*, was more resistant to 20 μg/mL INH than the wild-type strain transformed with the empty pMV261 vector (Fig. 1a). The evidence suggested that Ms0535 is potentially involved in regulating INH drug resistance in *M. smegmatis*. A sequence analysis revealed that Ms0535 contains an N-terminal winged HTH DNA-binding domain and a typical all-helical C-terminal domain belonging to the FadR subfamily [26]. The absence of the first α-helix indicates that Ms0535 encodes a VanR-like regulator (Fig. 1b).

### Ms0535 binds its own promoter

**Fig. 2:**
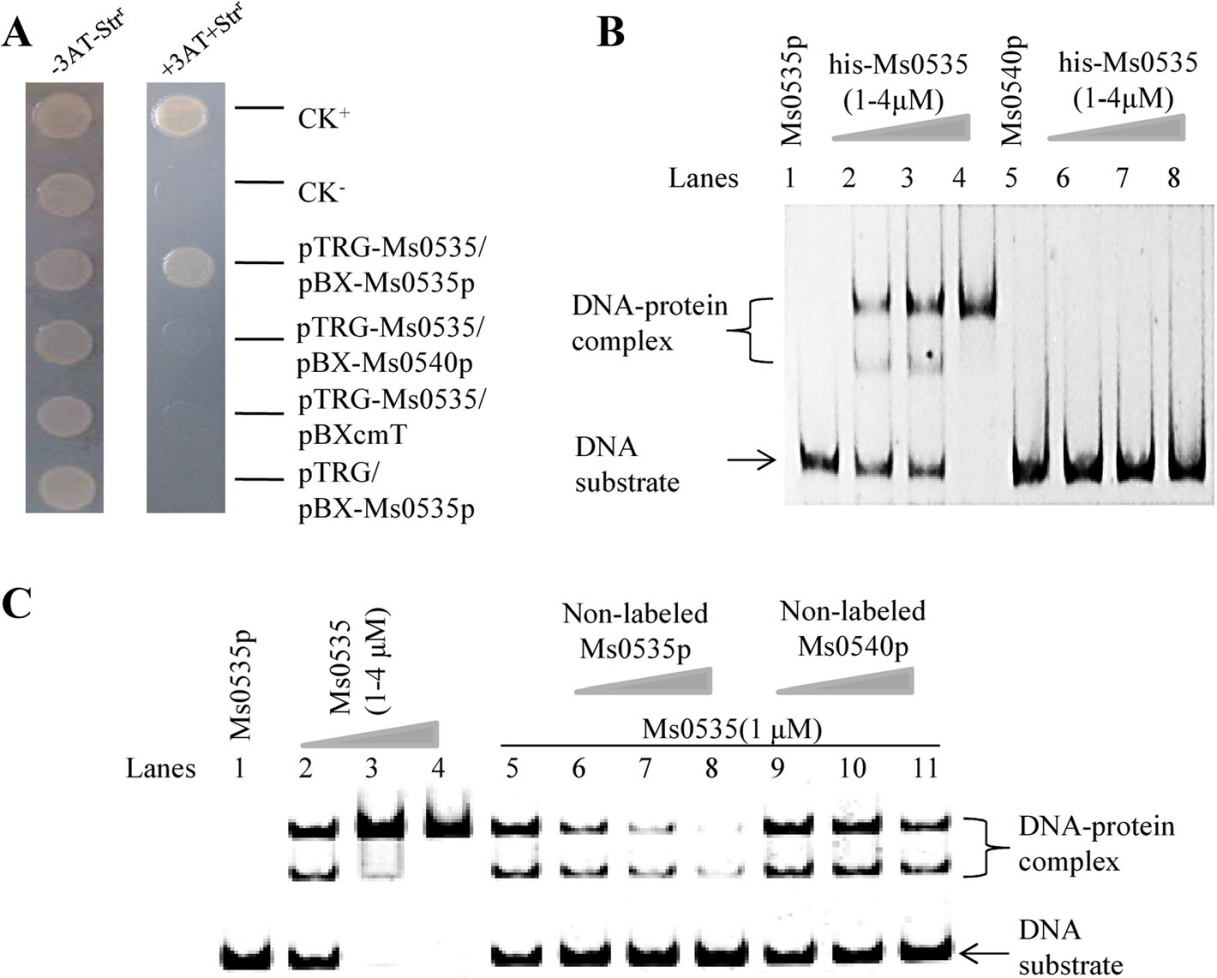
Ms0535 specifically binds to its own promoter. (**a**) Bacterial one-hybrid assays. Promoters of the *Ms0535* and *Ms0540* genes were cloned into the pBXcmT vector, and the *Ms0535* gene was cloned into the pTRG vector. A pair of pBXcmT/pTRG plasmids was co-transformed into the reporter strain, and then its growth was tested together with the self-activation controls on a selective medium. Co-transformants containing the pBX-*Rv2031*/pTRG-*Rv3133* plasmids [26] served as positive controls (CK+), and co-transformants containing the empty vectors pBXcmT and pTRG served as negative controls (CK−). (**b**) Electrophoretic mobility shift assays (EMSAs). The *Ms0535p* (lanes 1–4) and *Ms0540p* (lanes 5–8) DNA substrates were co-incubated with various amounts of the Ms0535 protein. The free DNA substrate and DNA-protein complexes are indicated. (**c**) EMSAs for the specific binding of Ms0535 to its own promoter. Then, 0.05 nM of fluorescein isothiocyanate-labeled *Ms0535* promoter DNA substrate was co-incubated with the Ms0535 protein in the absence (lanes 1–5) or presence of non-labeled *Ms0535p* (0.25-1 nM) (lanes 6–8) or non-labeled *Ms0540p* (0.25-1 nM) (lanes 9–11). Unlabeled *Ms0535* and unlabeled *Ms0540* promoter DNA substrates were used to compete with the labeled Ms0535 promoter DNA. The *Ms0535* promoter, but not the *Ms0540* promoter, inhibited the binding of Ms0535 to the labeled *Ms0535* promoter DNA substrate

Several assays were performed to detect the DNA binding activity of Ms0535. First, a bacterial one-hybrid system [27] was used to detected protein-DNA interactions. The promoter region (500 bp upstream) of *Ms0535*, *Ms0535p*, and the unrelated promoter *Ms0540p* (500 bp upstream of *Ms0540*) were cloned into the reporter vector pBXcmT [27] and co-transformed into reporter strains with pTRG-*Ms0535*. Co-transformants of both the positive control pTRG-*Rv3133c*/pBX-*Rv2031p*, as well as pTRG-*Ms0535*/pBX-*Ms0535p*, grew very well in the screening medium (Fig. 2a). In contrast, no growth was observed for the self-activated controls and the negative control (Fig. 2a). Thus, Ms0535 can bind to its promoter, *Ms0535p*.

Second, electrophoretic mobility shift assays (EMSAs) were conducted to show that Ms0535 binds to *Ms0535p in vitro.* As shown in Fig. 2b, 0.03 nM of the *Ms0535p* DNA substrate was co-incubated with different amounts (1–4 μM) of his-tagged Ms0535 protein, and clear band shifts were observed, indicating the formation of DNA-protein complexes (Fig. 2b, lanes 1–4). In contrast, Ms0535 did not bind to the *Ms0540p* negative control promoter (Fig. 2b, lanes 5–8). Additionally, a competition assay was performed. Unlabeled *Ms0535p* and *Ms0540p* promoters were used to compete with labeled *Ms0535p*, and *Ms0535p*, but not *Ms0540p*, could competitively inhibit the binding of Ms0535 to the labeled *Ms0535p* promoter (Fig. 2c).

**Fig. 3:**
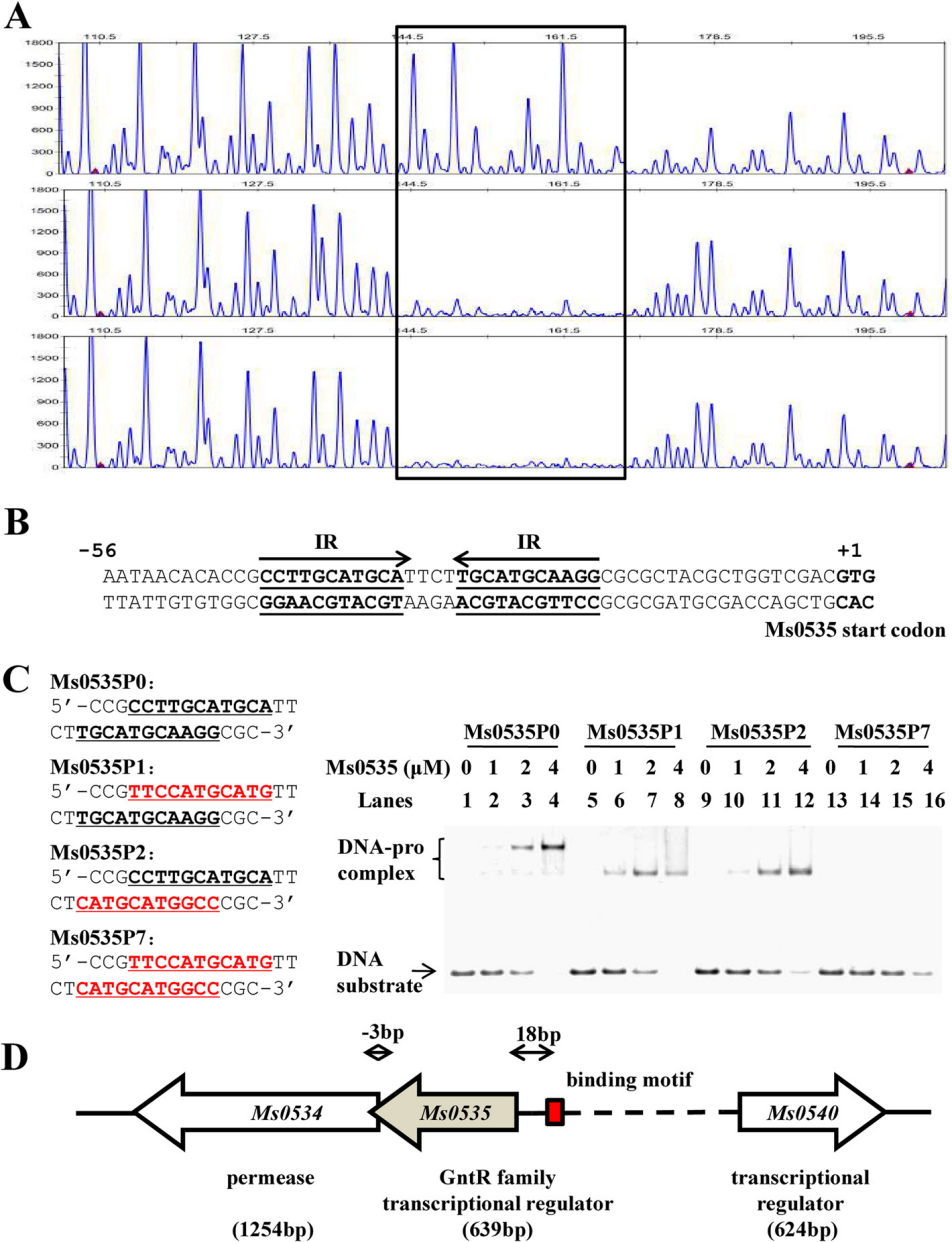
DNA-binding motif assays for Ms0535. (**a**) Dye primer sequencing based on a DNase I footprinting assay. The *Ms0535* promoter DNA was digested with DNaseI in the presence of increasing amounts of Ms0535. The protected regions are indicated by a black frame. (**b**) Sequence and structural characteristics of the protected *Ms0535* promoter region. The regions protected by Ms0535 are underlined. The binding motif is a 26-bp sequence containing invert repeats (IRs) with a 4-bp spacer. The translational start codon of *Ms0535* is indicated in bold. (**c**) Electrophoretic mobility shift assays of the DNA-binding activity of Ms0535 towards DNA substrates with (lanes 1–4) or without (lanes 13–16) the IR sequences. The DNA substrates were individually incubated with 0–4 μM of the Ms0535 protein. (**d**) Analysis of the domain structure of Ms0535 and the genomic location of its DNA binding motif. The *Ms0535* gene encodes a typical GntR regulator, and it shares a common upstream promoter region with the major facilitator superfamily permease Ms0534. The distance between the motif and the coding sequence of *Ms0535* is 18 bp

These findings strongly suggest that Ms0535 can specifically bind to its own promoter, *Ms0535p*.

### Ms0535 recognizes a palindromic sequence motif

To identify the DNA-binding site for Ms0535, a DNase I footprinting assay was performed as described previously [28]. The results showed that Ms0535 protected the sequence 5′-CCTTGCATGCATTCTTGCATGCAAGG-3′ in the *Ms0535p* promoter (Fig. 3a). The protected DNA region extended from positions −19 to −44 (assuming the Ms0535 start codon to be +1) in the coding strand (Fig. 3b). In this region, a palindromic motif formed by two IRs (5′-CCTTGCATGCA-3′), which are separated by 4 nt, was found (Fig. 3b). To detect binding between Ms0535 and the binding motif, short oligonucleotides were synthesized, and EMSAs were conducted. In the experiments, the wild-type motif and three mutants were included (Fig. 3c). The results showed that Ms0535 bound the wild-type *Ms0535p0* substrate, resulting in shifted bands (Fig. 3c, lanes 1–4). In addition, Ms0535 also bound to *Ms0535p1* and *Ms0535p2*, in which one IR was mutated (Fig. 3c, lanes 5–8 and 9–12, respectively). In contrast, Ms0535 did not bind the *Ms0535p7* fragment, in which both IRs were mutated (Fig. 3c, lanes 13–16). Interestingly, when Ms0535 bound to *Ms0535p1* and *Ms0535p2*, the single IR mutants, only one shifted band was observed (Fig. 3c, lanes 5–8 and 9–12, respectively), suggesting that Ms0535 may bind the promoter in two steps.

In conclusion, these results indicate that Ms0535 recognizes a 26-bp palindromic sequence motif.

### The *Ms0535* and *Ms0534* genes reside in an *Ms0534-0535* operon

**Fig. 4:**
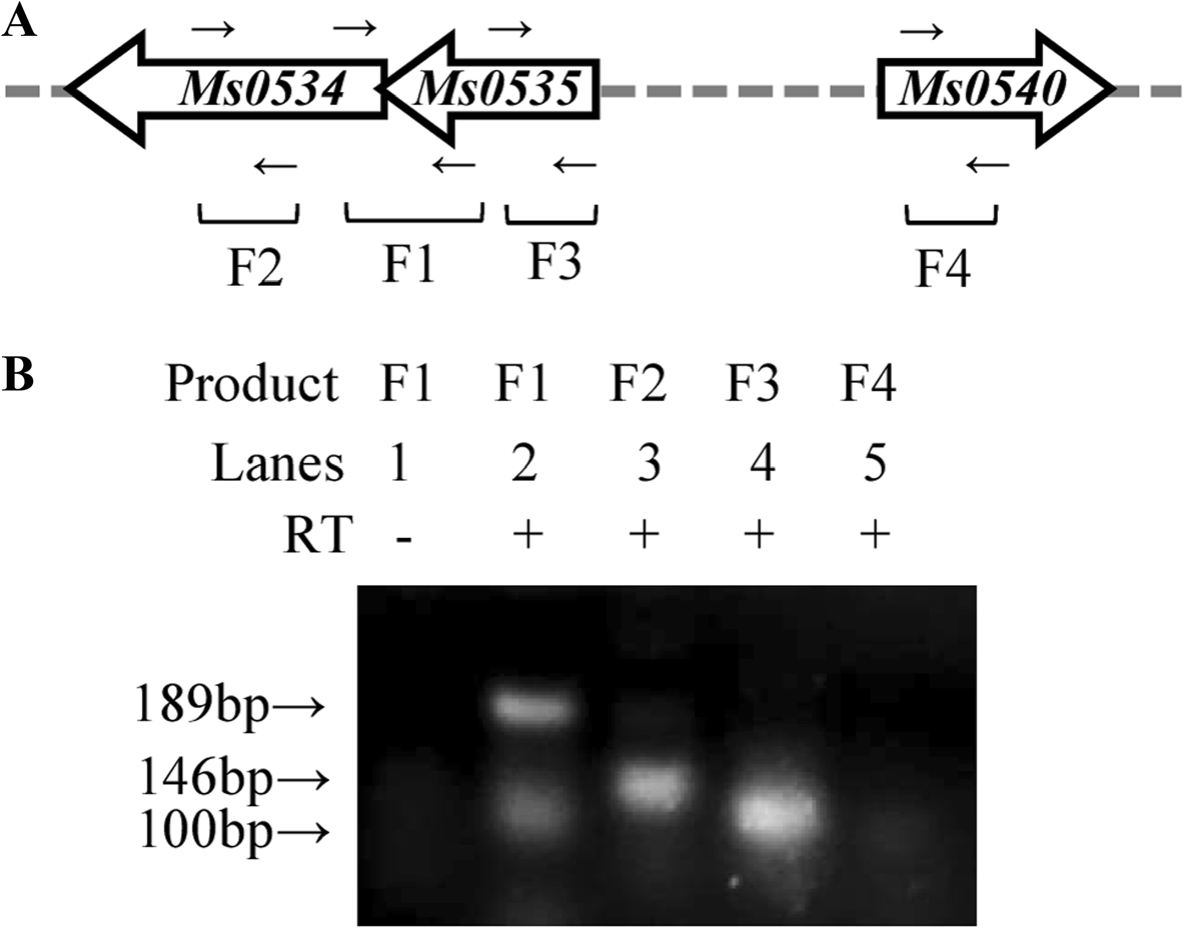
Assays for *Ms0534-Ms0535* co-transcription by reverse transcription-PCR. (**a**) The schematic operon structure of *Ms0534-Ms0535*. Primers designed for the assays are indicated by black arrows; F1 primers are complementary to the sequences of the two adjacent genes. (**b**) Reverse transcription-PCR assays for *Ms0534-Ms0535* co-transcription. In this assay, the resulting cDNA was transcribed by the F3 reverse primer. For the F1 fragment, mRNA (DNA-free) was used as a negative control (lane 1). In lane 2, a 189-bp reverse transcription-PCR product indicates the co-transcription of *Ms0534-Ms0535*. The 146-bp F2 and 100-bp F3 extension products are shown in lanes 2 and 3. The negative result in lane 5 reveals that the F4 fragment cannot be amplified from the cDNA template. The PCR procedure was as follows: the reactions underwent 35 cycles of denaturation at 95 °C for 30 s, annealing at 60 °C for 30 s, and extension at 72 °C for 30 s

Because Ms0535 binds to a 26-bp sequence motif in the upstream region of its own promoter, we created a system to locate the putative promoter and to detect the operator region of *Ms0535* (Fig. 3d). A genomic location analysis suggested that *Ms0535* shares the same upstream DNA region with the MFS permease Ms0534. Subsequent reverse transcription-PCR (RT-PCR) assays showed that the two genes were co-transcribed (Fig. 4). The cDNA used in this experiment was reverse transcribed by a reverse primer that was complementary to a region within the *Ms0535* gene. DNA fragments encompassing the *Ms0534* and *MS0535* genes, as well as the region between the genes, can be amplified from the cDNA template. An mRNA template and the amplification of an unrelated DNA fragment encompassing the *Ms0540* gene were used as negative controls. This result suggests that the *Ms0534* and *Ms0535* genes are co-transcripted and reside in an *Ms0534-Ms0535* operon in *M. smegmatis*.

### Ms0535 positively regulates its own expression and the expression of *Ms0534*

**Fig. 5:**
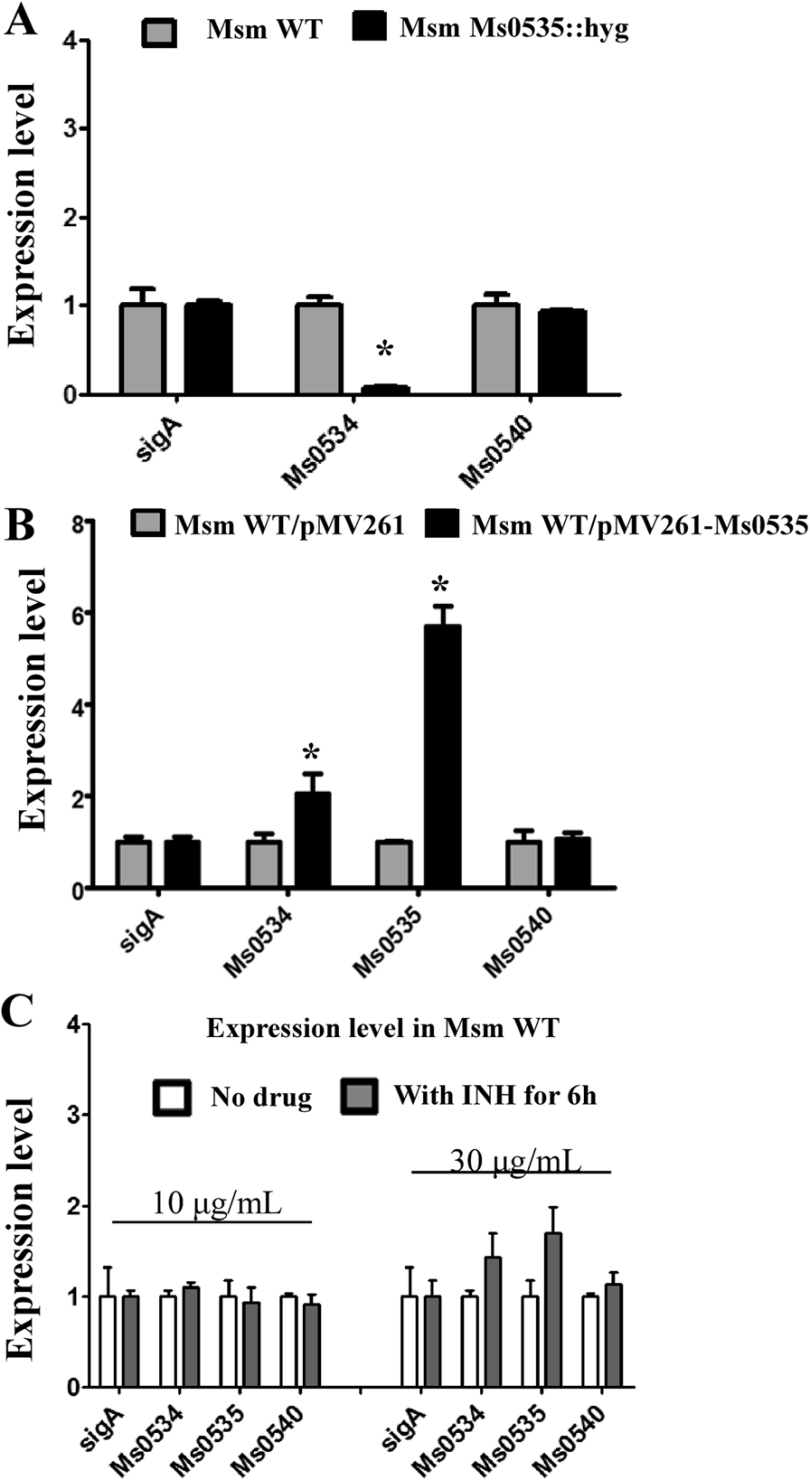
Quantitative real time-PCR assays. Gene expressions were determined in the *M. smegmatis Ms0535* deletion strain (**a**), in the *Ms0535*-overexpressing strain (**b**), and in wild type strain with or without isoniazid (**c**). The relative expression levels of the genes were normalized using the *sigA* gene as an invariant transcript. An unrelated promoter of the *Ms0540* gene was used as a negative control. Data were analyzed using the 2^**−ΔΔCt**^ method as described in the Methods. P-values of the relative expression data were calculated by an unpaired two-tailed Student’s *t*-test

To characterize the biological function of Ms0535, we constructed a *M. smegmatis Ms0535* knockout strain using a gene replacement strategy (Additional file 1: Figure S1). Subsequently, the expression of the adjacent gene, *Ms0534*, in both the wild-type and *Ms0535* deletion strains was compared by quantitative RT-PCR (qRT-PCR) assays. As shown in Fig. 5a, the expression of *Ms0534* was significantly down-regulated in the *Ms0535* knockout strain compared with that in the wild-type strain. In contrast, the expression of the negative control gene *Ms0540*, whose promoter does not contain a conserved binding motif, did not show any obvious changes. This finding suggested that Ms0535 could function as a positive regulator. Consistent with this view, when *Ms0535* was overexpressed (by approximately six-fold), the expression of *Ms0534* was significantly up-regulated (Fig. 5b). These results indicate that Ms0535 can function as a positive regulator of *Ms0535*/*Ms0534* in *M. smegmatis*.

As we proved, overexpressing Ms0535 can increase the INH resistance of *M. smegmatis*. To determine whether INH can affect the expression of *Ms0535*, qRT-PCR experiments were performed on the wild-type strain after cultivation in the presence or absence of INH for 6 h (Fig. 5c). Cultivation in the presence of 10 μg/mL INH did not alter the expression of the *Ms0534* and *Ms0535* genes. However, in the presence of 30 μg/mL INH, the expression of *Ms0535* increased by 1.4-fold relative to bacteria grown in the absence of INH, while *Ms0534* expression increased 1.6-fold. Since these differences in expression were not very significant, we concluded that these two genes are not INH-responsive genes, although overexpression of either Ms0535 or Ms0534 increases INH resistance (see below).

### Ms0535 regulates *M. smegmatis* antibiotic resistance via the putative permease Ms0534

*Ms0534* has been predicted to encode a permease that is possibly involved in drug export. Moreover, using the sequence of the Ms0535 DNA binding motif as a BLAST query, no more binding site was found in the *M. smegmatis* genome, except for the *Ms0535p* promoter. Taken together, we hypothesized that Ms0534 should be the effector protein that confers INH resistance in *Ms0535*-overexpressing strains. To test this hypothesis, *Ms0535* (Additional file 1: Figure S1) and *Ms0534* knockout strains (Additional file 2: Figure S2), together with multiple complementary strains, were constructed.

**Fig. 6:**
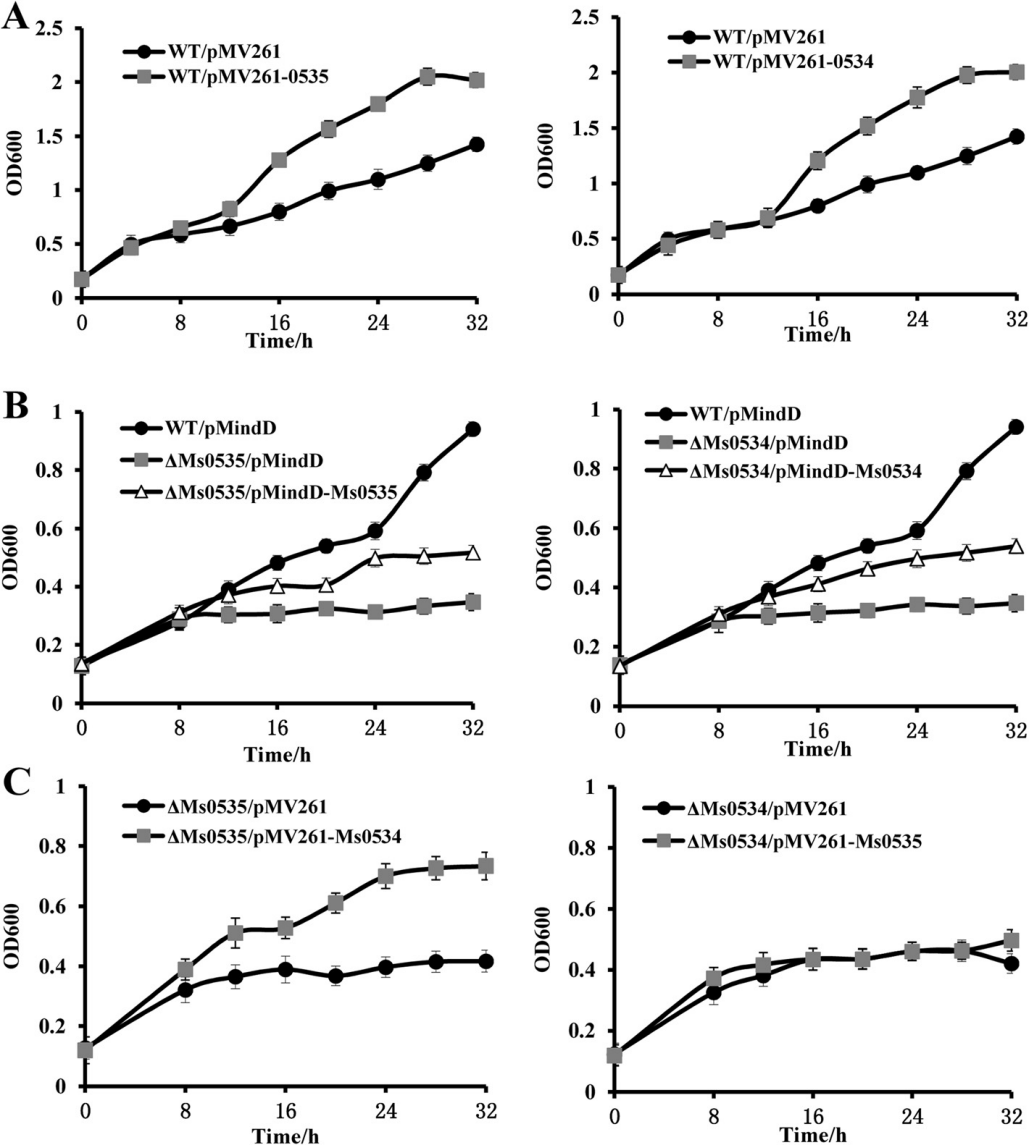
Determinations of growth curves of *M. smegmatis* strains*.* Mycobacterial strains were grown in 7H9 medium in the presence of 10 μg/ml isoniazid, and growth curves were determined. (**a**) Left: the wild-type (WT/pMV261) and *Ms0535*-overexpressing (WT/pMV261-*Ms0535*) strains; Right: the WT (WT/pMV261) and *Ms0534*-overexpressing (WT/pMV261-*Ms0534*) strains. To avoid potential side effects, the empty pMV261vector was included in the WT strain. (**b**) Left: the WT (WT/pMindD), *Ms0535* deletion (Δ*Ms0535*/pMindD), and Δ*Ms0535* complemented (Δ*Ms0535*/pMindD-*Ms0535*) strains; Right: the WT (WT/pMindD), *Ms0534* deletion (Δ*Ms0534*/pMindD), and Δ*Ms0534* complemented (Δ*Ms0534*/pMindD-*Ms0534*) strains. To avoid potential side effects, the empty pMindD vector was included in the WT and *Ms0534*/*Ms0535* deletion strains. (**c**) Left: the *Ms0535* deletion (Δ*Ms0535*/pMV261) strain and the Δ*Ms0535* strain complemented with *Ms0534* (Δ*Ms0535*/pMV261-*Ms0534*); Right: the *Ms0534* deletion strain (Δ*Ms0534*/pMV261) and the Δ*Ms0534* strain complemented with *Ms0535* (Δ*Ms0534*/pMV261-*Ms0535*). To avoid potential side effects, the empty pMV261 vector was included in the *Ms0534*/*Ms0535* deleted strains. Error bars represent the standard deviation of three biological replicates

We first determined the growth curves of the wild-type and *Ms0534*/*Ms0535*-overexpressing strains. As shown in Fig. 6a, the *Ms0535*-overexpressing strain grew much better than the wild-type strain in 7H9 medium containing 10 μg/ml INH (Fig. 6a, left panel), but their growth was similar in the absence of INH (Additional file 3: Figure S3A, left panel). Comparably, the *Ms0534*-overexpressing strain grew much better than the wild-type strain in the presence (Fig. 6a, right panel), but not in the absence of INH (Additional file 3: Figure S3A, right panel).

Furthermore, the growth curves of the wild-type, *Ms0535* deletion, and complemented strains were determined. As shown in Fig. 6b, both the *Ms0535* (left panel) and *Ms0534* deletion strains (right panel) were more sensitive to INH, and their sensitivity could be complemented by inducing the expression of *Ms0535* or *Ms0534*, respectively, using the pMindD system [17]. All these strains grew similarly in the absence of INH (Additional file 3: Figure S3B).

In addition, the *Ms0535* deletion strain was complemented with the *Ms0534* gene, and the *Ms0534* deletion strain was complemented with the *Ms0535* gene, and the growth curves of these strains were determined with (Fig. 6c) or without (Additional file 3: Figure S3C) INH. As shown in Fig. 6c, complementation with the *Ms0534* gene improved the growth of the *Ms0535* deletion strain (Fig. 6c, left). However, complementation with the *Ms0535* gene did not improve the growth of the *Ms0534* deletion strain (Fig. 6c, right).

These results support our hypothesis, and they indicate that Ms0535 regulates INH resistance through Ms0534, and Ms0534 is the effector protein that confers INH resistance to the *Ms0535*-overexpressing strain.

## Discussion

In recent decades, bacteria, especially some important pathogens such as *M. tuberculosis*, exhibiting drug and multi-drug resistance (MDR) have become a major health issue worldwide [29]. In the current study, we showed that a GntR family transcription factor, encoded by the *Ms0535* gene, activates the expression of the efflux pump protein Ms0534, an MFS permease, thereby affecting the drug resistance of *M. smegmatis*.

Drug efflux pump proteins play an important role in bacterial drug resistance [30, 31]. Several transcription factors involved in the regulation of these proteins have been characterized [6–8]. Ms2173, a global Cu^2+^-responsive GntR family transcription factor, was found to be involved in regulating the expression of 37 membrane transport genes, and it was shown to negatively affect mycobacterial drug resistance [18]. GntR family transcription factors, which can regulate MDR, antibiotic biosynthesis, cell permeability, and virulence [5, 32], are widely distributed in bacteria [26]; GntR is named after a repressor of the *B. subtilis* gluconate operon [5]. The family members usually function as transcriptional repressors, although there are also reports of GntR activators, such as *S. marcescens* PigT [18], *E. faecalis* CitO [19], and *B. subtilis* GabR [25]. The genomes of mycobacteria contain a large number of GntR family transcription factors [11], although little is known about the function of the other members of this family. In this study, we isolated and characterized the GntR/FadR subfamily transcriptional factor Ms0535, which acts as an activator and positively affects drug resistance in *M. smegmatis*. DNaseI footprinting assays and EMSAs confirmed that Ms0535 specifically recognized a 26-bp palindromic sequence motif, which was separated by 4 nt (Fig. 3a).

The MFS is one of the major types of bacterial drug efflux pumps, and it plays an important role in bacterial drug efflux [4, 5]. The current study showed that most of the mycobacterial drug efflux pumps belong to the MFS. This confirmed that the transporters of the MFS play an important role in mycobacterial drug resistance [33]. LfrA is a transporter protein that belongs to the MFS, and it is mainly involved in the efflux of EB and acriflavine in bacteria. It is the first drug efflux pump been discovered in *M. tuberculosis* [8]. Ms0534 is a permease that belongs to the MFS. It is located in the same operon as the GntR family transcriptional factor Ms0535. In this study, we found that Ms0535 activates the expression of Ms0534 (Fig. 5a)*.* An interesting finding from the present work was the determination that both Ms0535- and Ms0534-overexpressing mycobacterial strains exhibited higher levels of INH resistance than the wild-type strain (Fig. 6a left and right panels, respectively). Conversely, the *Ms0535* and *Ms0534* deletion strains were more sensitive to INH than the wild-type strain (Fig. 6a right panel; Fig. 6b, left panel). Furthermore, we found that overexpressing *Ms0534* in the *Ms0535* deletion strain enhanced mycobacterial INH resistance (Fig. 6b, middle panel). However, overexpressing *Ms0535* in the *Ms0534* deletion strain did not affect INH resistance (Fig. 6b, right panel). This suggests that Ms0535 acts as a transcriptional activator that regulates the expression of the drug efflux pump protein Ms0534, which positively regulates the drug resistance of *M. smegmatis.*

It is noteworthy that although the complementation strains grew better than the deletion strains under INH stress, their resistance to INH are still far from the wild-type control (Fig. 6b). One probable reason for this result is that the corresponding promoter activities are different in the pMindD vector and the *M. smegmatis* genome. Another possibility is that induce of *hyg* gene may cause polar effect in gene expression. Using a scarless gene deletion may avoid this effect by removing antibiotic resistance genes with phage infection [34].

As is well known, INH is active exclusively against mycobacteria, but pathogenic *M. tuberculosis* and non-pathogenic *M. smegmatis* display greatly different sensitivities to INH. Compared with its activity against *M. tuberculosis*, INH is 100 times less active against *M. smegmatis* [35]. Additionally, genomic variations between the two species may also be responsible for this difference. The *M. smegmatis* genome encodes more than 500 potential regulatory factors; however, only 214 regulatory factors are encoded by the *M. tuberculosis* genome. Ms0535 is a GntR family transcription factor encoded by the *M. smegmatis* genome, while absent in *M. tuberculosis*. Furthermore, its target Ms0534, a putative permease, is absent in *M. tuberculosis* as well. According to our results, these two genes may contribute the drug resistance of *M. smegmatis* and this partially explain the difference in INH resistance between the two mycobacterial species.

Studies have shown that MFS proteins can transport molecules such as drugs, glucide, and phosphate [5]. Consistently, we showed that overexpression of the transcription factor Ms0535 and the MFS permease Ms0534 affects the INH resistance of *M. smegmatis*. Additionally, no potential Ms0535 target genes were identified by searching the *M. smegmatis* with the sequence of the Ms0535 DNA binding motif. This suggests that Ms0534 may transport INH, and that Ms0535 specifically regulates this process.

## Conclusions

In the present study, a GntR family, VanR-like transcriptional factor, Ms0535, was shown to be a transcription activator and to positively regulate INH resistance of *M. smegmatis*. Ms0535 specifically recognized a 26-bp palindromic sequence motif separated by a 4-nt spacer, and it activated its own expression, as well as that of the *Ms0534* efflux pump gene in the same operon. Our findings establish Ms0535 as a novel GntR family activator in mycobacteria, and they significantly enhance our understanding of the regulatory mechanism of bacterial drug resistance.

## Methods

### Strains, enzymes, plasmids and reagents

*E. coli* BL21 cells and pET28a vector were purchased from Novagen (Darmstadt, Germany). pBT, pTRG vectors and *E. coli* XR host strains were purchased from Stratagene (La Jolla, CA, USA) (Additional file 4: Table S1). Restriction enzymes, T4 ligase, Modification enzymes, Pyrobest DNA polymerase, dNTPs and all antibiotics were obtained from TaKaRa Biotech (Shiga, Japan). The reagents for one-hybrid assay were purchased from Stratagene. Polymerase Chain Reaction (PCR) primers were synthesized by Invitrogen (Carlsbad, CA, USA) (Additional file 4: Table S2).

### The screening of isoniazid related transcriptional regulators

Over 500 transcriptional regulator genes were amplified from *M. smegmatis* genomic DNA. The gene fragments were mixed as a pool and cloned into pMV261 vector [36] to construct the regulatory genes overexpression plasmids library. The plasmids library were electrophoretic transferred into *M. smegmatis* mc2 155, and the strains were screened on 7H10 plates containing 20 μg/mL INH. As a result, those having increased INH resistance or decreased INH susceptibilities were identified as primary candidates. To avoid random mutations that may contribute to INH resistance, plasmid were extracted from each of the primary candidates, and transformed into the wild type *M. smegmatis* and assayed thrice in a similar way. In final, the increased INH resistance is sufficient to attribute to the overexpression of the corresponding transcriptional regulator.

### Bacterial one-hybrid assay

Bacterial One-Hybrid assays were carried out as described previously [27]. Ms0535 was cloned into the pTRG vector (Stratagene). Promoters (500 bp upsteam of the open reading frame) of the *M. smegmatis* genes were amplified using appropriate primers (Additional file 4: Table S2) and cloned into the pBXcmT vector. The selective plate contains 20 mM 3-AT (3-amino-1,2,4-triazole), 16 μg/ml streptomycin, 15 μg/ml tetracycline, 34 μg/ml chloramphenicol and 30 μg/ml kanamycin. A co-transformant containing pBX-Rv2031/pTRG-Rv3133 plasmids was served as positive control (CK+) and a co-transformant containing empty vector pBX and pTRG was served as negative control (CK-). A co-transformant containing pTRG-Ms0535/pBXcmT plasmids and pTRG/pBX-Ms0535p plasmids were served as self-activated controls. The plates were incubated at 30 °C for 3–4 days.

### Cloning, expression and protein purification

Cloning, expression and protein purification were performed as previously described [37]. *M. smegmatis* mc2 155 genes were amplified using specific primers (Additional file 4: Table S2). Ms0535 was cloned into the pET28a vector to produce recombinant vectors (Additional file 4: Table S1).

### Electrophoretic mobility shift assay (EMSA)

DNA fragments for the DNA-binding activity assays were amplified by PCR from *M. smegmatis* genomic DNA using their primers labeled with Fluorescein Isothiocyanateor (FITC) or directly synthesized (Additional file 4: Table S2). The EMSA assays were conducted as previously described [37].

### DNase I footprinting assay

The 300 bp promoter region of the Ms0535 gene (Additional file 4: Table S1) was amplified by PCR using appropriate primers labeled with Fluorescein Isothiocyanate (FITC) (Additional file 4: Table S2). The amplified products were purified with BioFlux PCR DNA Purification kit (BioFlux) and then subjected to similar binding reaction as in EMSA. DNaseI footprinting was performed using the method of dye primer sequencing [38, 39].

### Recombinant strains

Knockout of the Ms0535 and Ms0534 gene in *M. smegmatis* was performed as described previously [38, 40]. Deletion of Ms0535 and Ms0534 were further verified by Southern blotting [39]. Primers used in the assays were provided in Additional file 4: Table S1.

Ms0534 and Ms0535 genes were amplified from *M. smegmatis* genomic DNA by their respective primers (Additional file 4: Table S2). To overproduce in *M. smegmatis*, Ms0534 gene was inserted downstream of the hsp60 promotor of pMV261 [36]. And the Ms0534 and Ms0535 genes were cloned into a pMind vector [41] for complementing in knockout strains. The recombinant plasmids were electroporated into *M. smegmatis* mc2 155 and selected on 7H10 medium containing 30 mg/mL kanamycin.

### Quantitative real time PCR (qRT-PCR)

qRT-PCR were performed as described previously [38]. Isolation of mRNA and cDNA preparation of wild type strains, deletion mutants and overexpression *M. smegmatis* strains were performed and real-time PCR analysis was subsequently carried out according to previously described procedures [37]. Each PCR reaction (20 μl) contained 2 μl of 2 × SYBR Green Master Mix Reagent (Applied Biosystems), 1.0 μl of cDNA samples and 200 nM gene-specific primers (Additional file 4: Table S3). Gene expression levels were normalized to the levels of sigA rRNA gene transcripts and relative expression changes were calculated using the 2-ΔΔCt method [38, 42].

### Determination of mycobacterial growth curves

Growth patterns of the wild-type (Msm) mycobacterial strain, Ms0535-deleted mutant (Msm/Ms0535::hyg), Ms0534-deleted mutant (Msm/Ms0534::hyg), the overexpression (Msm/pMV261-Ms0535, Msm/pMV261-Ms0534) mycobacterial strains and complemented strains (Msm/pMindD-Ms0535, Msm/pMindD-Ms0534) were determined according to previously described [9].

## Availability of supporting data

The data sets supporting the results of this article are included within the article and its additional files.

## Additional files

Additional file 1: Figure S1. Construction of the *M. smegmatis Ms0535* knockout strain and Southern blotting assay. (A) Schematic of the recombination strategy for the deletion of the *Ms0535* gene from the *M. smegmatis* genome. (B) A map of the recombinant vector pMind-*Ms0535* containing the upstream and downstream sequences of *Ms0535* and the gene that confers resistance against hygromycin. (C) Schematic of the DNA fragments of the wild-type and Δ*Ms0535* knockout strains treated with the restriction enzyme SmaI. The probe is indicated with a black bar. (D) Southern blot assays. A 300-bp probe corresponding to the sequences of the *Ms0535* upstream genomic fragment of *M. smegmatis* was obtained by PCR and labeled with digoxigenin dUTP (Roche, Mannheim Germany). The probe was used to detect changes in the size of the SmaI-digested genomic fragment of *M. smegmatis* before and after recombination. (TIFF 942 kb) Additional file 2: Figure S2. Construction of the *M. smegmatis Ms0534* knockout strain and Southern blotting assays. (A) Schematic of the recombination strategy for the deletion of the *Ms0534* gene from the *M. smegmatis* genome. (B) A map of the recombinant vector pMind-*Ms0534* containing upstream and downstream sequences of *Ms0534* and the gene that confers resistance against hygromycin. (C) Schematic representation of the DNA fragments of the wild-type and Δ*Ms0534* knockout strains treated with the restriction enzyme NarI. The probe is indicated with a black bar. (D) Southern blot assays. A 300-bp probe corresponding to the sequences of the *Ms0534* upstream genomic fragment of *M. smegmatis* was obtained by PCR and labeled with digoxigenin dUTP (Roche). The probe was used to detect changes in the size of the NarI-digested genomic fragment of *M. smegmatis* before and after recombination. (TIFF 871 kb) Additional file 3: Figure S3. Growth curves of *M. smegmatis* strains in the absence of isoniazid. The strains are the same as those described in Fig. 6. (TIFF 228 kb) Additional file 4: Table S1. Strains and plasmids used in this study. **Table S2.** Primers used in this study. **Table S3.** Primers used for quantitative real time-PCR and reverse transcription PCR. (DOC 67 kb)

## Abbreviations

3-AT: 3-amino-1,2,4-triazole
qRT-PCR: Quantitive real-time PCR
ABC: ATP-binding cassette superfamily
EB: Ethidium bromide
EMB: Ethambutol
EMSA: electrophoretic mobility shift assay
FITC: Fluorescein Isothiocyanateor
INH: Isoniazid
MFS: Major facilitator superfamily
MATE: Multidrug and toxic compound extrusion family
MDR: Multi-drug resistance
RIF: Rifampicin
RND: Resistance/nodulation/cell division family
SMR: Small multidrug resistance family
