# Supplementary figures and images for: A GntR family transcription factor positively regulates mycobacterial isoniazid resistance by controlling the expression of a putative permease
Source: BMC Microbiol. 2015 Oct 16;15:214. doi: 10.1186/s12866-015-0556-8 (PMC4609117; doi:10.1186/s12866-015-0556-8)

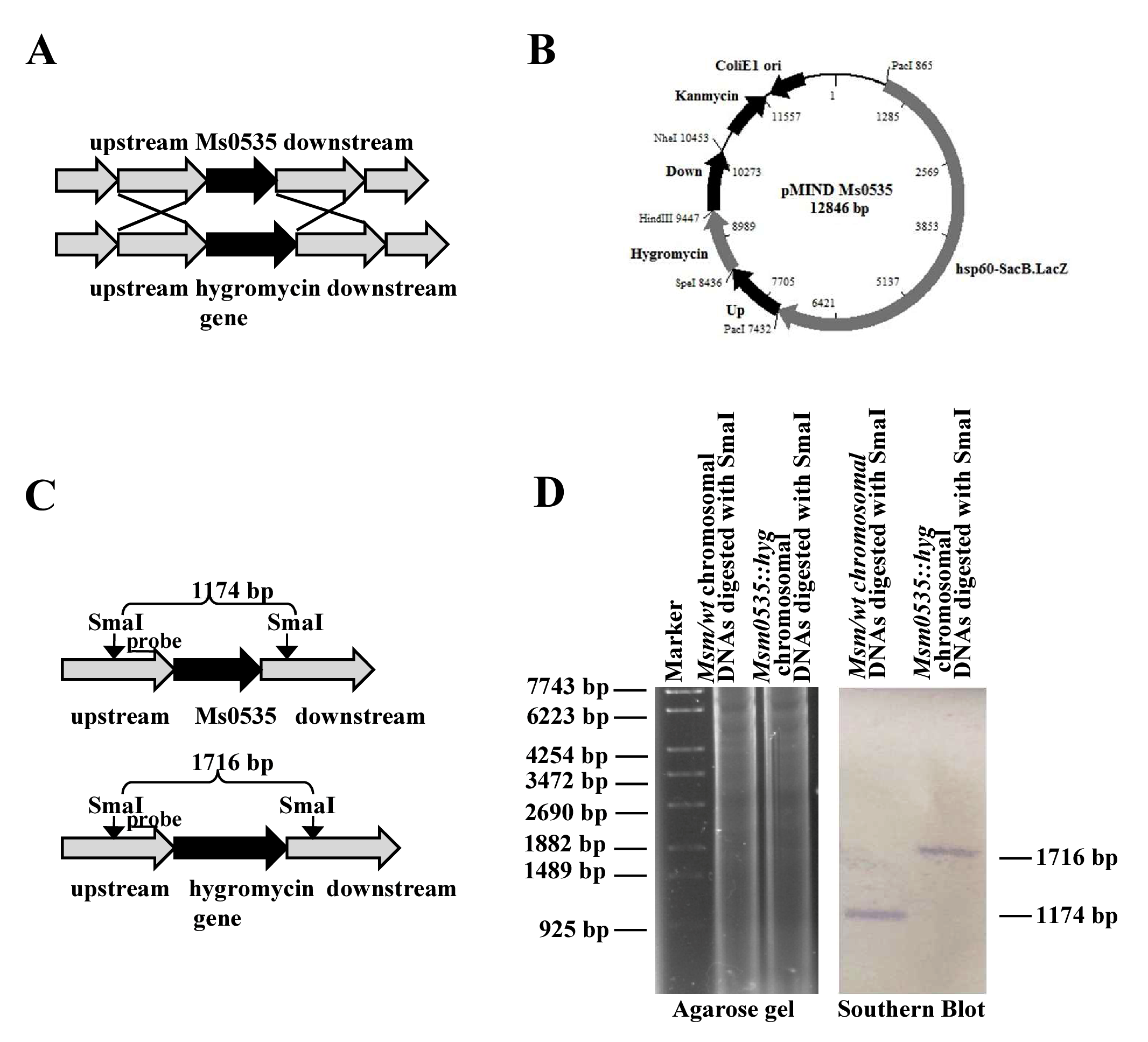

Supplement: Additional file 1: Figure S1. — Construction of the M. smegmatis Ms0535 knockout strain and Southern blotting assay. (A) Schematic of the recombination strategy for the deletion of the Ms0535 gene from the M. smegmatis genome. (B) A map of the recombinant vector pMind-Ms0535 containing the upstream and downstream sequences of Ms0535 and the gene that confers resistance against hygromycin. (C) Schematic of the DNA fragments of the wild-type and ΔMs0535 knockout strains treated with the restriction enzyme SmaI. The probe is indicated with a black bar. (D) Southern blot assays. A 300-bp probe corresponding to the sequences of the Ms0535 upstream genomic fragment of M. smegmatis was obtained by PCR and labeled with digoxigenin dUTP (Roche, Mannheim Germany). The probe was used to detect changes in the size of the SmaI-digested genomic fragment of M. smegmatis before and after recombination. (TIFF 942 kb) [file 12866_2015_556_MOESM1_ESM.tif]

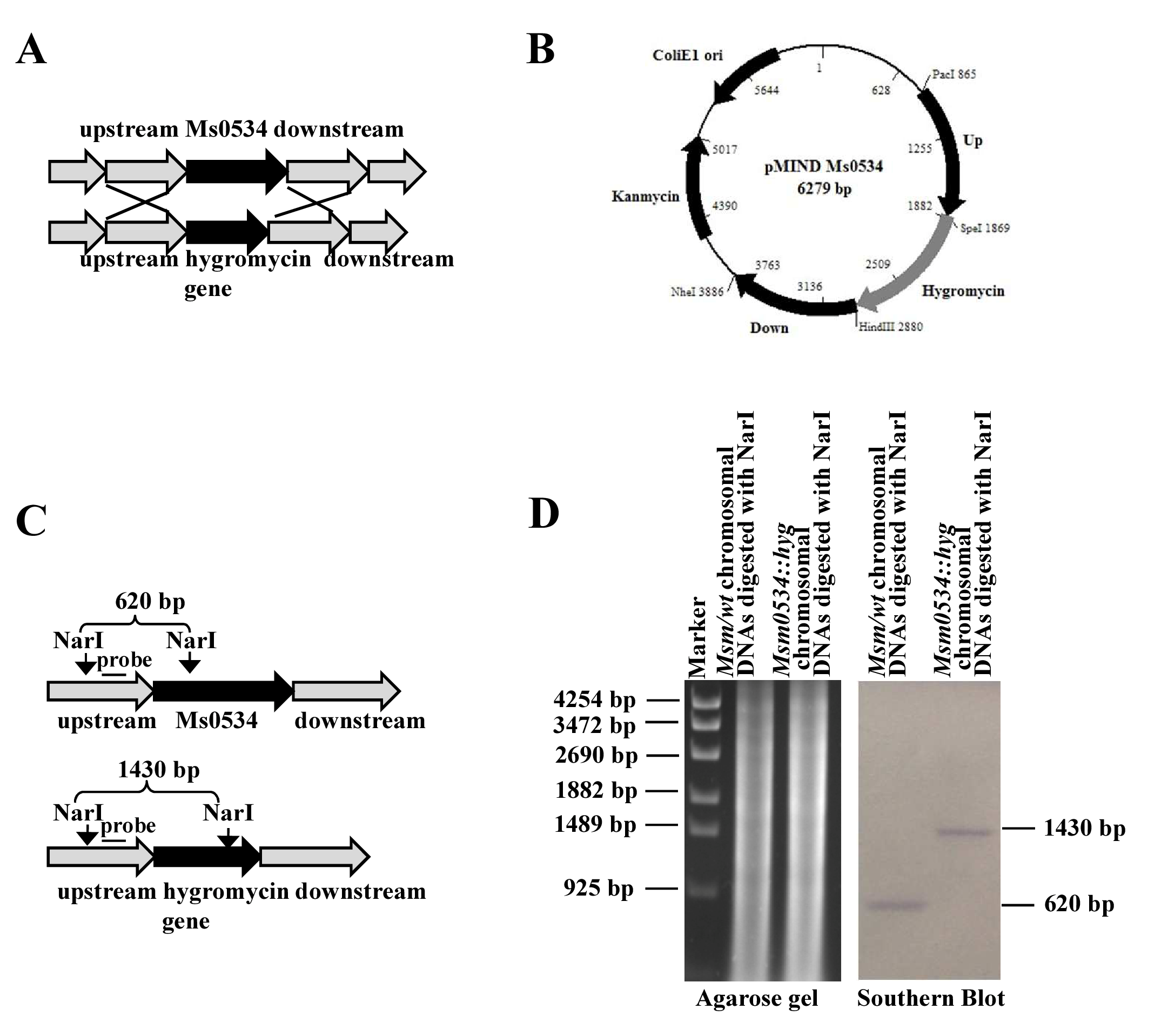

Supplement: Additional file 2: Figure S2. — Construction of the M. smegmatis Ms0534 knockout strain and Southern blotting assays. (A) Schematic of the recombination strategy for the deletion of the Ms0534 gene from the M. smegmatis genome. (B) A map of the recombinant vector pMind-Ms0534 containing upstream and downstream sequences of Ms0534 and the gene that confers resistance against hygromycin. (C) Schematic representation of the DNA fragments of the wild-type and ΔMs0534 knockout strains treated with the restriction enzyme NarI. The probe is indicated with a black bar. (D) Southern blot assays. A 300-bp probe corresponding to the sequences of the Ms0534 upstream genomic fragment of M. smegmatis was obtained by PCR and labeled with digoxigenin dUTP (Roche). The probe was used to detect changes in the size of the NarI-digested genomic fragment of M. smegmatis before and after recombination. (TIFF 871 kb) [file 12866_2015_556_MOESM2_ESM.tif]

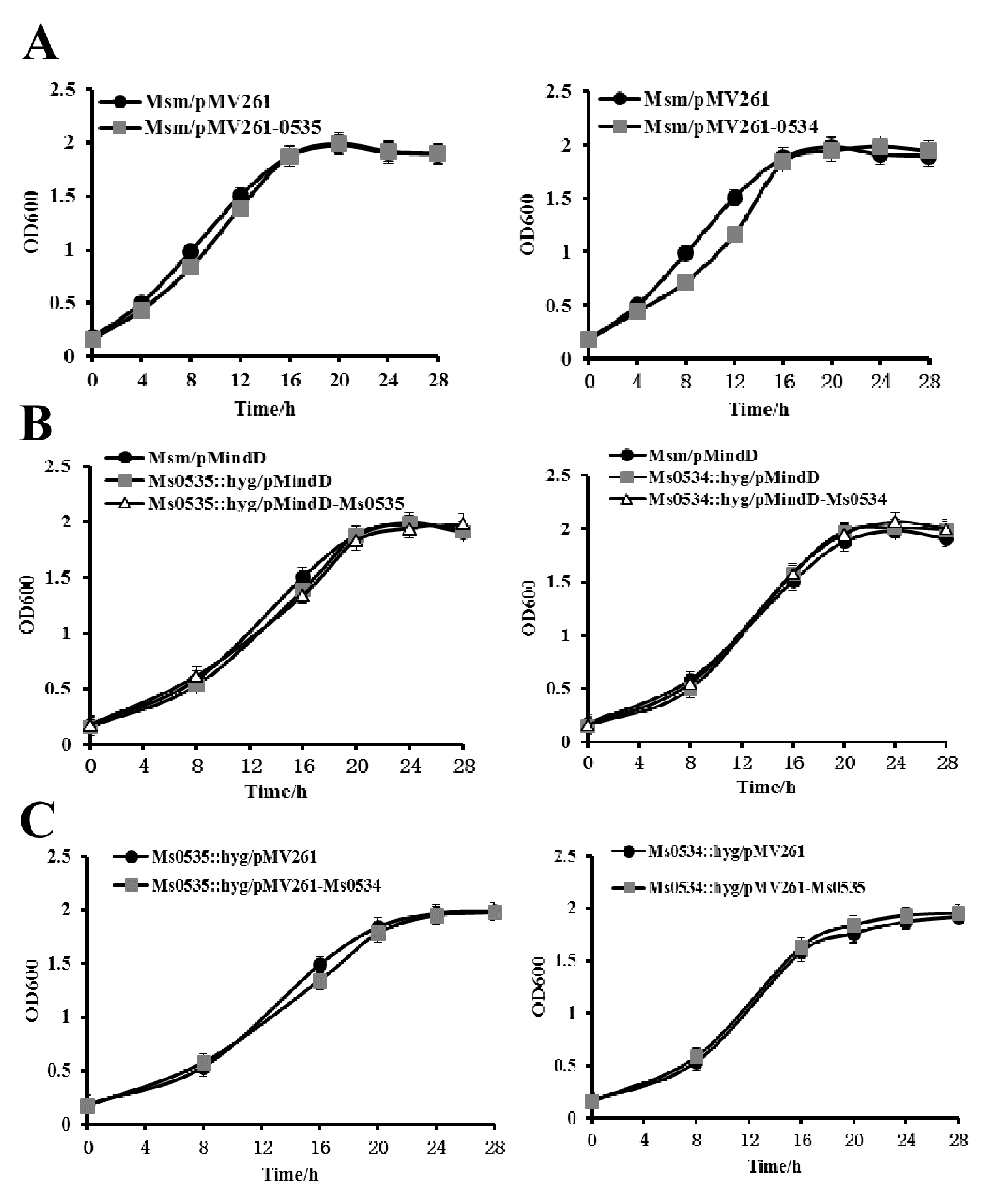

Supplement: Additional file 3: Figure S3. — Growth curves of M. smegmatis strains in the absence of isoniazid. The strains are the same as those described in Fig. 6. (TIFF 228 kb) [file 12866_2015_556_MOESM3_ESM.tif]
