# Supplementary material for: A GntR family transcription factor positively regulates mycobacterial isoniazid resistance by controlling the expression of a putative permease
Source: BMC Microbiol. 2015 Oct 16;15:214. doi: 10.1186/s12866-015-0556-8 (PMC4609117; doi:10.1186/s12866-015-0556-8)
Supplement: Additional file 4: Table S1. — Strains and plasmids used in this study. Table S2. Primers used in this study. Table S3. Primers used for quantitative real time-PCR and reverse transcription PCR. (DOC 67 kb) [file 12866_2015_556_MOESM4_ESM.doc]

**Table S1 Strains and plasmids used in this study**

| Plasmid or Strain | Relevant genotype or features | Source or reference |
| --- | --- | --- |
| Strain  *E.coli* |  |  |
| DH5a | Host for plasmid construction | TaKaRa |
| BL21 | Host for overexpression | TaKaRa |
| XR | Host for bacteria one-hybrid | Stratagene |
| *M. smegmatis mc^2^155* |  | 8 |
| Msm/WT | *M. smegmatis* | 8 |
| Msm/pMV261 | mc^2^155 with pMV261 | This study |
| Msm/pMV261-Ms0535 | mc^2^155 with pMV261::Ms0535 | This study |
| Msm0535::hyg | mc^2^155 gene *Ms0535* replaced by *hyg* | This study |
| Msm0535::hyg /pMV261 | Msm0535::*hyg* with pMV261 | This study |
| Msm/pMV261-Ms0534 | mc^2^155 with pMV261::*Ms0534* | This study |
| Msm0534::hyg | mc^2^155 gene *Ms0534* replaced by *hyg* | This study |
| Msm0534::hyg/pMV261  △Z1  △Z2  △Z3  △Z4  Z1  Z2  Z3  Z4 | Msm0534::*hyg* with pMV261  Msm0535::*hyg* with pMV261::GroEl1::*lacZ*  Msm0535::*hyg* with pMV261::Ms0535p::*lacZ*  Msm0535::*hyg* with pMV261:: Ms0540p::*lacZ*  Msm0535::*hyg* with pMV261::*lacZ*  mc^2^155 with pMV261::GroEl1::*lacZ*  mc^2^155 with pMV261::Ms0535p::*lacZ*  mc^2^155 with pMV261:: Ms0540p::*lacZ*  mc^2^155 with pMV261::*lacZ* | This study  This study  This study  This study  This study  This study  This study  This study  This study |
| Plasmid  pET28a(+) | Kan^r^, lacZ operon, T7 promotor, His-Tag | Novagen |
| pET-Ms0535 | Ms0535 in *EcoR*I-*Xba*I of pET28a | This study |
| pBXcmT | Chlo, p15A replicon, lac-UV5 promoter | 19 |
| pBX-Ms0535p | Ms0535p in *EcoR*I-*Xba*I of pBXcmT | This study |
| pBX-Ms0540p | Ms0540p in *EcoR*I-*Xba*I of pBXcmT | This study |
| pTRG | Tet, ColE1 replicon, lpp/lac-UV5 promoter | Stratagene |
| pTRG-Ms0535 | Ms0535p in *EcoR*I-*Xba*I of pTRG | This study |
| pMV261 | Kan^r^, pAL5000 replicon | 8 |
| pMV261::GroEl1::lacZ  pMV261::Ms0535p::lacZ  pMV261:: Ms0540p::lacZ  pMV261::lacZ | GroEL1 in *EcoR*I -*Hind*Ⅲ of pMV261  Ms0535p in *EcoR*I-*Xba*I of pMV261  Ms0540p in *EcoR*I-*Xba*I of pMV261  lacZ in *Hind*Ⅲ-*Nhe*I of pMV261 | This study  This study  This study  This study |
| pMV261-Ms0535 | Ms0535 in *EcoR*I-*Xba*I of pMV261 | This study |
| pMindD- Ms0535 | Ms0535 in *EcoR*I-*Xba*I of pMindD | This study |
| pMV261- Ms0534  pMindD- Ms0534 | Ms0534 in *EcoR*I-*Xba*I of pMV261  Ms0534 in *EcoR*I-*Xba*I of pMindD | This study  This study |

**Table S2 Primers used in this study**

| Name | Sequence5’-3’ | Usage |
| --- | --- | --- |
| Ms0535f  Ms0535r  Ms0534f  Ms0534r  Ms0535upf  Ms0535upr  Ms0535dnf  Ms0535dnr  Ms0534upf  Ms0534upr  Ms0534dnf  Ms0534dnr  Ms0535pf  Ms0535pr  Ms0540pf  Ms0540pr  Ms0535fpf  Ms0535fpr-2  Ms0535P0f  Ms0535P0r  Ms0535P1f  Ms0535P1r  Ms0535P2f  Ms0535P2r  Ms0535P7f  Ms0535P7r  Ms0535TZf  Ms0535TZr  Ms0534TZf  Ms0534TZr | AGTCATGAATTCAGGTGGACACGACCAGCAAC  GCGCGCTCTAGATCATTTCAAGGTAACCCGAT  TTATGAATTCTGATGACCGCCACAGCAACACC  TATTTCTAGATCACACCCTGGCCGTGACGG  CCGCTTAATTAACGCCACATGATCCGGATGGC  TATTACTAGTGTCGACCAGCGTAGCGCGCCTT  GCTGAAGCTTATGACCGCCACAGCAACACC  TAATGCTAGCATGCCGTATGCGCAGCCGAG  TCACTTAATTAAGCGTTCGTCCACGACTTCGT  GCCTACTAGTTCATTTCAAGGTAACCCGATAG  TAATAAGCTTCCCGACGAATCGGGTACCTC  TAATGCTAGCAGGTTCGTCCAGATCAGCTG  AGATGAATTCGCAGACCGGCTCGATCGACG  ATATTCTAGAGTCGACCAGCGTAGCGCGCC  ATATGAATTCTGCGCAGCACCTTGCGGTGC  AGATTCTAGAGGGACACGCTCCATCACTCG  ACGGTGCTGGACGAGGAGGT（FITC标记）  ACTGAGCAGTTGGCCACCGC  CACACCGCCTTGCATGCATTCTTGCATGCAAGGCGCGCTA  Synthesized directly TAGCGCGCCTTGCATGCAAGAATGCATGCAAGGCGGTGTG Synthesized directly  CACACCGTTCCATGCATGTTCTTGCATGCAAGGCGCGCTA Synthesized directly  TAGCGCGCCTTGCATGCAAGAACATGCATGGAACGGTGTG Synthesized directly  CACACCGCCTTGCATGCATTCTCATGCATGGCCCGCGCTA Synthesized directly  TAGCGCGGGCCATGCATGAGAATGCATGCAAGGCGGTGTG Synthesized directly  CACACCGTTCCATGCATGTTCTCATGCATGGAACGCGCTA Synthesized directly  TAGCGCGTTCCATGCATGAGAACATGCATGGAACGGTGTG Synthesized directly  GTCCACGACTTCGTGAACTC  AATGCATGCAAGGCGGTGTG  ACGTCGTCGACTCGCTGTTC  AGGGTGGACTCGAACTCCTC | Clone and expression Clone and expression  Clone and expression  Clone and expression  Knock out  Knock out  Knock out  Knock out  Knock out  Knock out  Knock out  Knock out  Clone, EMSA  Clone, EMSA  Clone, EMSA  Clone, EMSA  EMSA  EMSA  EMSA  EMSA  EMSA  EMSA  EMSA  EMSA  EMSA  EMSA  Southern blotting  Southern blotting  Southern blotting  Southern bloting |

Notes: Restriction enzyme sites are underlined.

**Table S3 Primers for quantitative real-time PCR (qRT-PCR) and reverse transcribed PCR (RT-PCR)**

| Name | Sequence 5’-3’ | Usage |
| --- | --- | --- |
| Ms0535RTf (F3f)  Ms0535RTr (F3r)  Ms0534RTf (F2f)  Ms0534RTr (F2f)  Ms0540RTf (F4f)  Ms0540RTr (F4r)  sigARTf  sigARTr  F1f  F1r | AGGCATTCCTGCGGTTGG  AGGAGGTCGGTGCCCTCTTG  TCTGCCTGTCGCTCGGTCTC  TTCGATGGTGCGGTGCCGGATG  TATCCCACCAAGGCCGAGAC  CCAGATCGAGCAGCCAGTCC  CGAGGAAGAAGAAGCTGATG  CGTCTTTGCGTGCCTGTC  TCATCGGTGAACACGCCCGCCT  TTGCCGCCCCAACCGATGCAGAAG | qRT-PCR, RT-PCR  qRT-PCR, RT-PCR  qRT-PCR, RT-PCR  qRT-PCR, RT-PCR  qRT-PCR, RT-PCR  qRT-PCR, RT-PCR  qRT-PCR  qRT-PCR  RT-PCR  RT-PCR |
